# Mathematical Modeling of Targeted Drug Delivery Using Magnetic Nanoparticles during Intraperitoneal Chemotherapy

**DOI:** 10.3390/pharmaceutics14020324

**Published:** 2022-01-29

**Authors:** Mohsen Rezaeian, M. Soltani, Ahmad Naseri Karimvand, Kaamran Raahemifar

**Affiliations:** 1 Department of Mechanical Engineering, K. N. Toosi University of Technology, Tehran 19967-15433, Iran; mohsenrezaeian@email.kntu.ac.ir (M.R.); ahmad.Naserik999@gmail.com (A.N.K.); 2 Centre for Biotechnology and Bioengineering (CBB), University of Waterloo, Waterloo, ON N2L 3G1, Canada; 3 Department of Electrical and Computer Engineering, University of Waterloo, Waterloo, ON N2L 3G1, Canada; 4 Advanced Bioengineering Initiative Center, Multidisciplinary International Complex, K. N. Toosi University of Technology, Tehran 14176-14411, Iran; 5 Data Science and Artificial Intelligence Program, College of Information Sciences and Technology (IST), Penn State University, Pennsylvania, PA 16801, USA; kvr5517@psu.edu; 6 Department of Chemical Engineering, University of Waterloo, 200 University Avenue West, Waterloo, ON N2L 3G1, Canada; 7 School of Optometry and Vision Science, Faculty of Science, University of Waterloo, 200 University Avenue West, Waterloo, ON N2L 3G1, Canada

**Keywords:** intraperitoneal chemotherapy, magnetic drug targeting, peritoneal carcinomatosis, computational oncology, targeted drug delivery

## Abstract

Intraperitoneal (IP) chemotherapy has emerged as a promising method for the treatment of peritoneal malignancies (PMs). However, microenvironmental barriers in the tumor limit the delivery of drug particles and their deep penetration into the tumor, leading to reduced efficiency of treatment. Therefore, new drug delivery systems should be developed to overcome these microenvironmental barriers. One promising technique is magnetically controlled drug targeting (MCDT) in which an external magnetic field is utilized to concentrate drug-coated magnetic nanoparticles (MNPs) to the desired area. In this work, a mathematical model is developed to investigate the efficacy of MCDT in IP chemotherapy. In this model, considering the mechanism of drug binding and internalization into cancer cells, the efficacy of drug delivery using MNPs is evaluated and compared with conventional IP chemotherapy. The results indicate that over 60 min of treatment with MNPs, drug penetration depth increased more than 13 times compared to conventional IPC. Moreover, the drug penetration area (DPA) increased more than 1.4 times compared to the conventional IP injection. The fraction of killed cells in the tumor in magnetic drug delivery was 6.5%, which shows an increase of more than 2.5 times compared to that of the conventional method (2.54%). Furthermore, the effects of magnetic strength, the distance of the magnet to the tumor, and the magnetic nanoparticles’ size were evaluated. The results show that MDT can be used as an effective technique to increase the efficiency of IP chemotherapy.

## 1. Introduction

Cancers that occur in organs associated with the peritoneal cavity are prone to metastasize to the intraperitoneal space. Patients with these malignancies experience low quality of life due to the fact of complications such as urinary blockage, ascites, pain, and inability to eat and drink. The five-year survival rate is 49% in these types of cancer. The average survival rate in peritoneal carcinomatosis of gastric origin is 1–3 months [1]. Management of peritoneal metastases (PMs) has always been a challenging issue. Chemotherapy with systemic injections in the 1980s had a palliative approach and predicted a survival time of less than a few months. With the introduction of peritoneal plasma membrane as an opportunity for the regional treatment of PMs by IP injection of chemotherapy drugs, the treatment of these patients entered a new phase. The new treatment had promising results, to the extent that a combination of hyperthermic IP chemotherapy along with cytoreductive surgery (CRS) have led to a longer survival time and complete cure in some cases [1,2,3,4]. Although IPC has shown a promising treatment outcome for patients with PM [5], generally poor drug penetration into the tumor has prevented widespread clinical use of this method [6]. IP administration delivers large amounts of anticancer drugs to the intraperitoneal space. Thus, tumors are subjected to a large proportion of these drugs [7]. The unique pathophysiology of the tumor, including a denser extracellular matrix (ECM), ineffective lymphatic system, leaky and spatial heterogeneous vasculature, and an increased interstitial fluid pressure, prevents the drug from effectively penetrating the tumor. In addition, the side effects of chemotherapy limit the dose of the administered drug. In this case, finding a way to concentrate the drug only in the tumor area can be helpful. Various drug delivery strategies have been studied for this purpose [8,9,10,11,12,13]. Meanwhile, magnetically controlled drug targeting (MCDT) is one of the promising techniques that has been used for more than two decades [14]. In this method, an external magnetic field facilitates the drug-coated magnetic nanoparticle (MNPs) targeting. This method has traditionally been studied to overcome the blood flow and concentrate MNPs around the blood vessel in systematic injection [10,15,16,17,18,19,20].

While some studies have shown that magnetic drug targeting in biological fluids is strongly hampered [21], IP chemotherapy using MNPs could be an alternative for intravenous administration in the treatment of peritoneal carcinomatosis by directly delivering the MNPs to the tumor tissue. In a magnetically controlled IP drug targeting system, MNPs are injected into the peritoneal cavity and directed by an external magnetic to the tumor site. Then, these particles can be diffused into the tumor tissue. Drug particles can bind to the tumor cells and finally become internalized into the cancer cells [22]. Successful drug delivery depends on several different parameters such as the number of nanoparticles delivered to the tumor, the penetration depth of the particles into the tumor, and the rate of binding affinity to cancer cells. Nanoparticle delivery is controlled by size-dependent microenvironmental parameters such as the relative size of these particles compared to the vessel wall pore size and ECM pore size [23,24]. There is also a competition between drug diffusion in the tumor tissue and drug binding to adjacent cancer cells. The high rate of drug binding to adjacent cancer cells can affect their penetration to more distant cells in the tumor [25]. Hence, the study of magnetic drug delivery by IP injection requires considering all of the abovementioned aspects of the transport process.

In this work, we used a numerical model to study the IP injection of drug-coated MNPs for the treatment of peritoneal malignancies. Size dependency was seen as a determining parameter, taking into account the impacts of the relative size of MNPs compared to the vessel wall pore size and ECM pore size in the model. Moreover, the drug transport process was examined by considering the mechanisms of drug binding and internalization into cancer cells. The spatiotemporal distribution of drug concentration in the forms of free, bound, and internalized cancer cells are presented. Finally, the fraction of killed cells (FK) was calculated as a quantitative treatment efficacy parameter.

## 2. Materials and Methods

**Figure 1:**
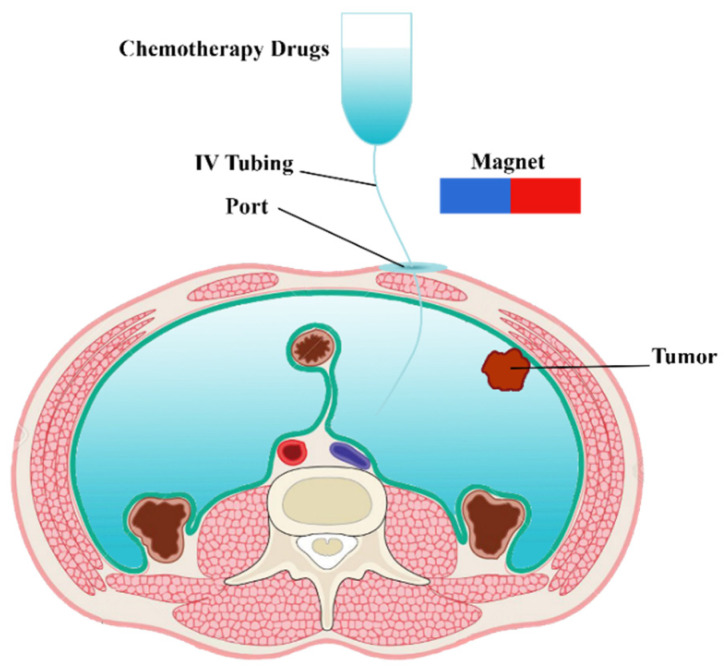
Schematic of a magnetically controlled IP drug targeting system.

In clinical IP chemotherapy, chemotherapeutic agents are transferred to the desired location within a 1–2 h cycle. A schematic of drug delivery by IP injection of MNPs is shown in Figure 1. Drug-coated MNPs are injected into the peritoneal cavity and guided by a permanent magnet. In the following, the governing equations of a magnetically controlled IP drug targeting system are presented.

### 2.1. Interstitial Fluid Flow

Considering the intercapillary distance (33–98 μm), which is usually 2–3 orders of magnitude smaller than the length scale of drug transfer [26,27], tumor tissue acts as a porous media. Hence, Darcy’s law in porous media was used to explain the interstitial fluid flow [28,29]:

(1) $$v_{i}=-\kappa \nabla P_{i}$$

Here, *κ* is the interstitium’s hydraulic conductivity. In addition, *P_i_* and $v_{i}$ are the interstitial fluid pressure and velocity, respectively. The steady-state mass conservation equation for the incompressible interstitial fluid is as follows:

(2) $$\nabla .v_{i}=\phi_{B}-\phi_{L}$$

In this equation, $\phi_{B}$ is the net flow rate from blood vessels into the interstitium per unit volume, and $\phi_{L}$ is the net lymphatic drainage per unit volume. Starling’s law was used to calculate $\phi_{B}$ and $\phi_{L}$ according to the equation below:

(3) $$\phi_{B}=\frac{L_{PL}S}{V}\left(P_{B}-P_{i}-\sigma_{S}\left(\pi_{B}-\pi_{i}\right)\right)$$

where *L_P_* and $\frac{S}{V}$ indicate the hydraulic conductivity of the microvascular wall and vascular surface area per unit volume, respectively, and $P_{B}$ and $P_{i}$, are the intravascular pressure and interstitial pressure, respectively. $\sigma_{s}$ and *π_B_* indicate the average osmotic reflection coefficient of plasma proteins and plasma osmotic pressure. Likewise, *π_i_* is the interstitial fluid osmotic pressure. Lymphatic drainage ($\phi_{L})$ is related to the difference between interstitial fluid pressure and lymphatic pressure:

(4) $$\phi_{L}=\frac{L_{PL}S_{L}}{V}\left(P_{i}-P_{L}\right)$$

where *L_PL_* is the hydraulic conductivity of the lymphatic vessel wall, $\frac{S_{L}}{V}$ is the surface area per unit volume of lymphatic vessels, and $P_{L}$ is the pressure in the lymphatic vessels. Here, $\phi_{L}$ is considered zero because of the lack of an effective lymphatic system in solid tumors.

### 2.2. Mass Transport

Maxwell–Ampere’s law was used to correlate the magnetic field and the current intensity based on the magneto-static nature of the problem (Equation (5)). Moreover, Gauss’s law (Equation (6)) was used to describe the magnetic flux density [18,30]:

(5) ∇×H=J

(6) ∇.B=0

Here, *H* is the magnetic field, *J* is the current intensity, and *B* is the magnetic flux density. As we are using a permanent magnet, the current density (*J*) was considered 0. For the air and tissue domain, the constitutive equation *B* = *μ*_0_*H* was used, while for the magnet domain, the equation *B* = *μ*_0_*μ_r_* *H* + *B_rem_* was applied [31]. In these equations, the vacuum magnetic permeability was considered *μ*_0_ = 4 π × 10^−7^ NA^−2^, the relative magnetic permeability *μ_r_* = 1000, and the remnant magnetic flux was defined as *B_rem_*. In a magnetic field (*H*), the magnetic force exerted on an MNP is determined as [32]:

(7) $$F_{m}=V_{MNP}\frac{\mu_{0}\chi }{1+\chi /3}{\left[\frac{\partial \vec{H}}{\partial \vec{x}}\right]}^{T}\vec{H}=\frac{1}{2}V_{MNP}\frac{\mu_{0}\chi }{1+\chi /3}\nabla ({\vec{\left\|H\right\|}}^{2})$$

Here, $V_{MNP}=\frac{4}{3}\pi a^{3}$ and χ are the volume and magnetic susceptibility of MNPs, respectively. In the case of a strong magnetic field, which can cause particle saturation [33], ${\left[\frac{\partial \vec{H}}{\partial \vec{x}}\right]}^{T}\vec{H}$ is replaced by ${\left[\frac{\partial \vec{H}}{\partial \vec{x}}\right]}^{T}{\vec{M}}_{sat}$, where ${\vec{M}}_{sat}$ indicates the saturated magnetization of the particle [33,34,35]. For well-made particles, $\left\|{\vec{M}}_{sat}\right\|$ is on the order of 0.5 T and lines up with $\vec{H}$ [36,37]. The saturation of the particle does not affect the direction of the force, only its magnitude.

The free drug’s concentration in the interstitial fluid is calculated using the convection–diffusion equation as follows:

(8) $$\frac{\partial C_{F}}{\partial t}=-v\nabla C_{F}+D_{mnp}\nabla^{2}C_{F}-\frac{1}{\varphi }K_{ON}C_{rec}C_{F}+K_{OFF}C_{B}+\Phi$$

where *C_F_* is the free drug concentration, *D_F_* is the diffusion coefficient of free drug in the porous medium, and *C_rec_* and *φ* are the concentration of cell surface receptors and the tumor volume fraction available for the drugs, respectively. *K_ON_* is the constants of the drug association rate to the cancer cells. In addition, *v* and *D_mnp_* are the velocity and effective diffusion coefficient of MNPs in tissue. The velocity (*v*) was calculated as the sum of the local velocity of interstitial fluid (*v_i_*) and the equilibrium velocity (*v_e_*). *v_e_* corresponds to when the Stokes drag force, *Fs* = (6*πaη*)*ve*, is equal to the magnetic force (*Fm*) [15,18]:

(9) $$v_{e}=\frac{F_{m}}{6\pi a\eta }$$

Here, *η* = 1.12 × 10^−3^ Pa.s is the dynamic viscosity of the interstitial fluid.

*Φ* in Equation (8), is the net rate of free drug exchange from blood and lymphatic vessels and is calculated as follows:

(10) $$\Phi =\Phi_{B}-\Phi_{L}$$

Using the pore model [38,39] for trans-capillary transfer, *Φ_B_* and *Φ_L_* are expressed as follows:

(11) $$\Phi_{B}=\left(\phi_{B}\left(1-\sigma_{f}\right)C_{P}+\frac{PS}{V}\left(C_{P}-C\right)\frac{Pe}{e^{Pe}-1}\right)-\phi_{L}C$$

(12) $$\Phi_{L}=\phi_{L}C$$

where *P* is the permeability of tumor microvessels, *Cp* is the concentration of MNPs in the blood plasma, and *σ_f_* is the osmotic reflection coefficient for the MNPs. *Pe* is the Peclet number defined as follows:

(13) $$Pe=\frac{\phi_{B}\left(1-\sigma_{f}\right)}{P\frac{S}{V}}$$

The equation for the bound drug concentration is as follows:

(14) $$\frac{\partial C_{B}}{\partial t}=\frac{1}{\varphi }K_{ON}C_{rec}C_{F}-K_{OFF}C_{B}-K_{INT}C_{B}$$

where *C_B_* is the drug concentration bound to the cancer cell, and *K_INT_* is the constant rate of drug internalization into the cancer cells. Moreover, the equation internalized drug concentration is expressed as:

(15) $$\frac{\partial C_{I}}{\partial t}=K_{INT}C_{B}$$

where *C_I_* is the concentration of the drug that is internalized into cancer cells.

### 2.3. Cell Survival Model

The fraction of killed cells was calculated as $1-S_{F}$, where $S_{F}$ is the fraction of surviving cells. $S_{F}$ was obtained from Equation (16) [40], which is based on an in vitro study by Ker et al. [41] for the fraction of non-small surviving lung tumor cells.

(16) $$S_{F}=exp\left(-10^{6}\cdot \omega C_{I}\right)$$

In Equation (16), ω=0.4938 and $C_{I}$ indicates the internalized drug. The values of the model parameters, including tissue and solute transport parameters, are listed in Table 1 and Table 2, respectively.

### 2.4. Model Geometry and Boundary Conditions

**Figure 2:**
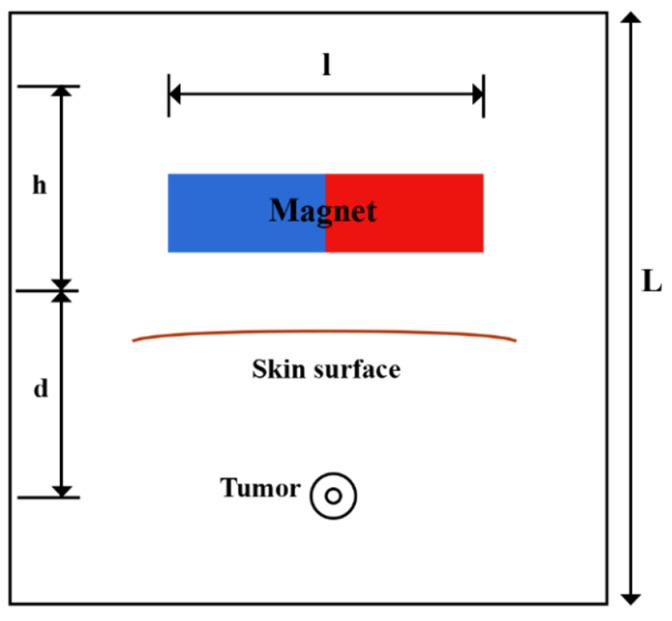
Schematic of the geometry of the magnetically controlled IP drug targeting model.

A solid tumor may have a necrotic area that lacks functional blood or lymph vessels; thus, there is no exchange of fluid with the interstitium in this region. In contrast, the regions of the tumor that are outside of the necrotic core contain rapidly dividing cells and blood vessels. In order to consider this non-uniform perfusion rate in the tumor, a biologically related structure, including an area with a necrotic core and a surrounding with leaky vasculature, is defined as a tumor [49]. A representation of the model’s geometry is presented in Figure 2. A magnet with length, *l*, and width, *h*, lies at a distance, *d*, from the tumor. A circular tumor with radius, R, and a necrotic core with radius, R_n_, were considered. Doxorubicin has been used as one of the most widely used drugs in the model.

The boundary conditions for the present study are listed in Table 3. For internal boundaries in the tumor, the continuity of IFP, concentration, and its flux were considered, where $\Omega^{-}$ and $\Omega^{+}$ demonstrate the necrotic and viable areas of the tumor at the boundary. Moreover, for the outer boundary, the Dirichlet boundary condition was applied.

### 2.5. Solution Strategy

**Figure 3:**
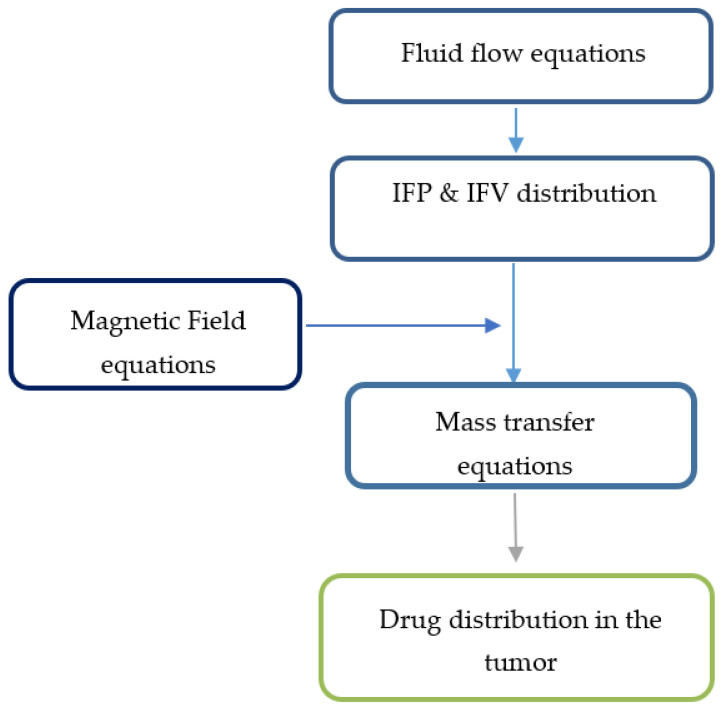
Solution strategy for the magnetically controlled IP drug targeting model.

To create the initial conditions for time-dependent numerical simulations, fluid flow equations were primarily solved to achieve a steady-state solution in the computational domain. Then, the values of velocity and pressure obtained at time zero were used to simulate drug delivery. The solution strategy for this section is also shown in Figure 3.

## 3. Results and Discussion

### 3.1. Conventional IPC

In this section, we examine the results of drug transfer by conventional IP chemotherapy. The spatiotemporal distribution of the free drug (*C_F_*), bound drug (*C_B_*), and drugs internalized into cancer cells (*C_I_*) were examined in the tumor during one hour of chemotherapy. Two main criteria were considered for the evaluation of the performance of chemotherapy with this method:

1. Fraction of killed cells (*FK*) was utilized as the major parameter for quantitatively evaluating drug delivery efficiency. Since drug particles do not reach all tumor parts in chemotherapy, this fraction was first studied as the fraction of killed cells in the drug penetration area (*FK_PA_*). Then, in order to evaluate the treatment of the whole tumor, this parameter was calculated and examined as the effective fraction of killed cells (*FK_eff_*), which represents the fraction of killed cells by considering the whole tumor;
2. To evaluate the performance of the drug delivery system in improving the penetration of the drug into the tumor, *w*_1/2_ is defined as the distance from the outer border of the tumor where the concentration of a free drug is equal to 50% of the concentration at the tumor border [50]. This parameter considers the slope of decreasing the drug concentration by moving from the tumor border to its depth. The larger the value of *w*_1/2_, the lower the concentration drop when moving to the depth of the tumor and the greater the penetration of the drug into the tumor.

#### 3.1.1. Distribution of IFV and IFP in the Tumor

**Figure 4:**
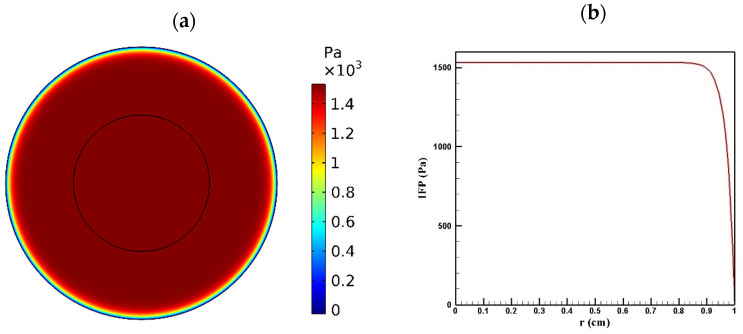
IFP distribution in a tumor with a radius of 10 mm: (**a**) contour of IFP distribution in the tumor; (**b**) IFP distribution values along the tumor radius.

**Figure 5:**
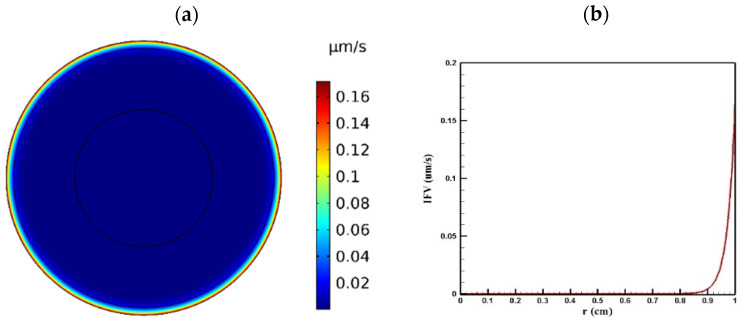
IFV distribution in a tumor with a radius of 10 mm: (**a**) contour of IFV distribution in the tumor; (**b**) IFV distribution values along the tumor radius.

Tumor microenvironment features have a crucial role in the way a drug is delivered to the tumor. Higher cell density in tumors results in reducing the permeability of tumor tissue compared to the normal one. Figure 4 and Figure 5 show the distribution of IFP and IFV in the tumor, respectively. As indicated in Figure 4a,b, IFP was highest at the tumor center (1533 Pa), except in a small area near the outer boundary of the tumor where the pressure decreased significantly. According to Darcy’s law (Equation (1)), since the pressure gradient is zero in a large part of the tumor, IFV had an insignificant value in this area (Figure 5a). Similarly, in the outer boundary of the tumor, because of the high amount of pressure gradient, the IFP increased sharply and reached its maximum level (0.17 μm/s) (Figure 5b).

#### 3.1.2. Concentration Distribution in the Tumor

**Figure 6:**
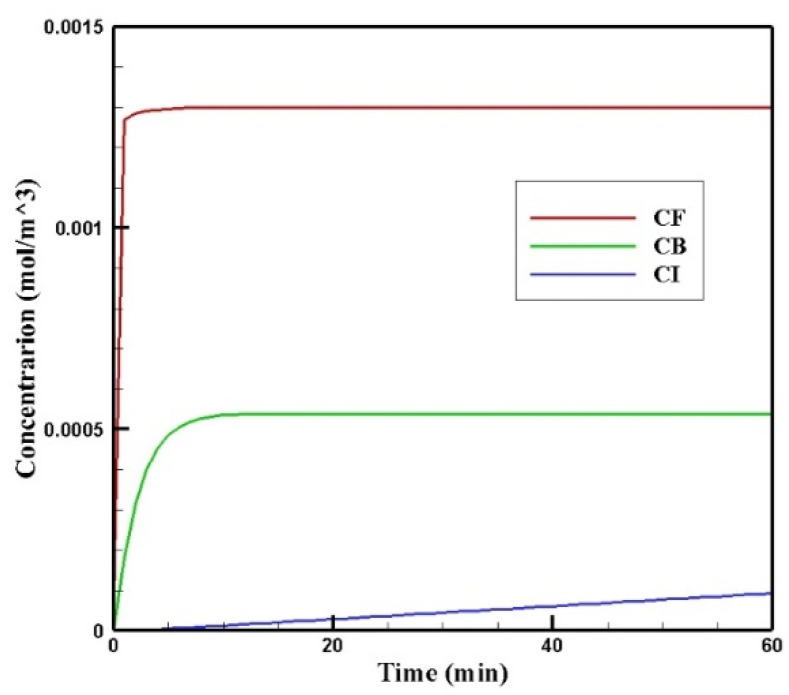
Mean concentrations of the free drug (*C_F_*), bound drug (*C_B_*), and internalized drug (*C_I_*) versus time during 60 min of conventional IPC.

Figure 6 shows the profiles of the average concentration of doxorubicin in the forms of free, bound, and internalized over 60 min of injection time. As is clear, immediately after injection, the concentration of free doxorubicin in the tumor skyrocketed to its maximum level (0.0013 mol/m^3^) and then plateaued. The same applied to the concentration of bound drug, but the difference was that the concentration of bound drug reached its maximum more gradually. Unlike free and bound drug concentrations, the concentration of internalized drug was constantly increasing. This increasing internalized drug concentration cell as well as the non-decreasing trend of the free and bound drugs were due to the constant concentration of the drug at the outer boundary of the tumor within one hour of administration. In other words, with the continuous injection of free drug, the concentration of free and bound drug reached a steady-state during treatment. Obviously, with the end of the drug injection into the peritoneal cavity, the mentioned concentrations will decrease. However, the main focus in this study was a one-hour time span in which a continuous concentration of the drug was injected into the peritoneal cavity.

**Figure 7:**
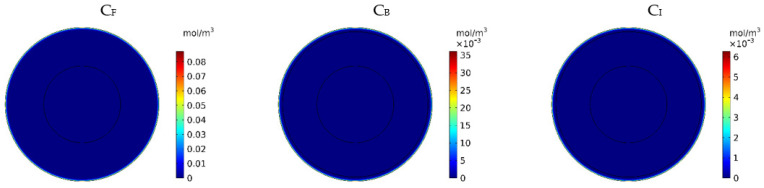
Contours of the free, bound, and internalized drug concentrations after 60 min of treatment.

**Figure 8:**
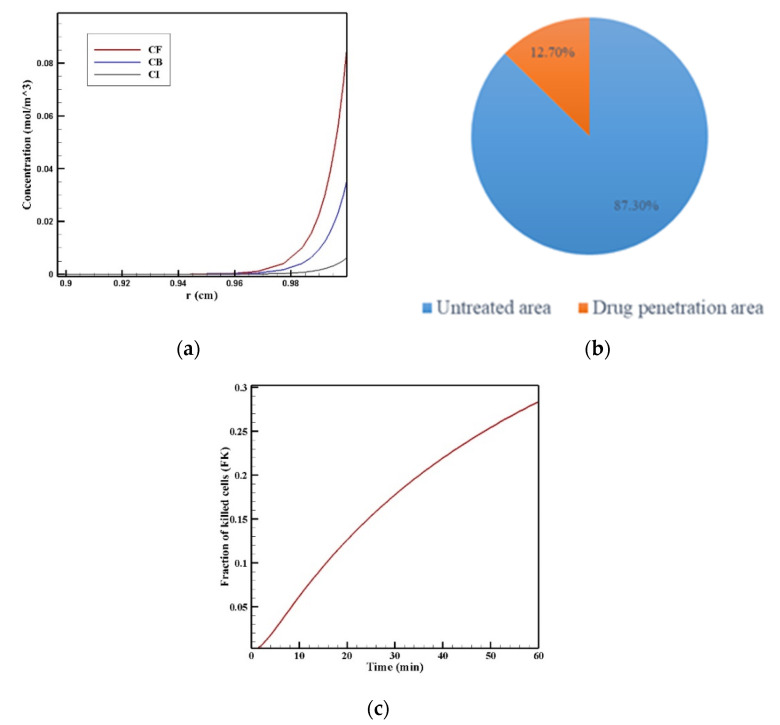
(**a**) Mean concentration profiles of the free drug (*C_F_*), bound drug (*C_B_*), and internalized drug (*C_I_*) along the tumor radius after 60 min of treatment; (**b**) pie chart of the drug penetration area and untreated area of the tumor; (**c**) fraction of killed cells in the drug penetration area (*FK_PA_*) in terms of time over 60 min of chemotherapy by IP injection.

Figure 7 show the contours of the concentration of free, bound, and internalized drugs with the IP injection of doxorubicin at one hour after injection. As seen in Figure 7, the penetration of the drug into the tumor by IP chemotherapy was curbed to a limited area of the outer boundary of the tumor and, as a result, a large part of the tumor was out of reach for drug delivery. This can be examined more precisely in Figure 8a, where the mean drug concentration profiles in the tumor for the free, bound, and internalized forms along the tumor radius after 60 min of treatment are presented. As shown in this figure, the concentration decreased rapidly to zero as they moved away from the tumor boundary. Since the internalized drug was supplied by the drug that was bound to the cancer cells and the bound drug was also a part of the free drug available in the tumor, the concentration of the internalized drug was therefore always much lower than the free drug concentration available in the tumor.

In order to acquire a higher concentration of the drug internalized into the cancer cells, more extracellular drugs should be available. Moreover, the drug must penetrate the tumor at the proper depth so that the tumor is homogeneously exposed to the drug. For this reason, the *w*_1/2_ was examined to evaluate the drug’s penetration into the tumor during treatment. The value obtained for *w*_1/2_ in this section was equal to 60 μm and, given that the tumor’s radius was 10 mm, the relative penetration depth of the tumor *w*_1/2_% was obtained as 0.6%. In addition, calculating the relative drug penetration area (*PA_rel_*) revealed that 87.3% of the tumor area remained untreated (Figure 8c). These outcomes accurately show one of the main challenges of the IPC, which is the very low penetration depth of the drug. The poor drug penetration depth was a result of the adverse pressure gradient at the tumor boundary followed by the outward convection flow, which exists at the tumor boundary as described in Figure 4 and Figure 5. In order to evaluate the effectiveness of treatment during 60 min of chemotherapy by IP injection, the time profile of the fraction of killed cells (*FK*) in the drug penetration area is presented in Figure 8c. The *FK_PA_* values increased over 60 min of treatment and reached 28.3% at the end of treatment.

Table 4 summarized the calculated values for the treatment evaluation parameters, including drug penetration depth in tumor *w*_1/2_, the relative drug penetration area (*PA_rel_*), fraction of killed cells in the area of drug penetration (*FK_PA_*), and the effective fraction of killed cells (*FK_eff_*) after 60 min of treatment. According to this table, *PA_rel_* and *FK_eff_* are 8.97% and 2.54%, respectively.

The results of this section generally indicate the low efficacy of the conventional IPC. Even though the IP injection has been conspicuous because of its potential in the regional treatment and direct transfer of drugs to the tumor, it is necessary to provide strategies that can improve treatment using IP injection. The results presented in this section provide a general insight into drug delivery by conventional IP injection and review it from the perspective of fluid and mass transfer, which can help design a treatment strategy that can overcome obstacles of drug delivery to the tumor.

### 3.2. Magnetically Controlled IP Drug Targeting

In this section, the results of the MNP delivery are presented. To evaluate the improvement of treatment using this method. The results of this section were compared to the results of conventional IPC. Firstly, a baseline model is presented in order to compare magnetic IP drug delivery with the conventional IPC. Next, the impact of the MNPs’ size, the magnetic strength, and the distance of the magnet from the tumor were investigated.

#### 3.2.1. Baseline Model

In the baseline model, a permanent magnet of 20 by 10 cm in dimension, which was placed at a distance of 5 cm from the tumor, was used to assist in drug delivery to the tumor. The radius of the MNPs used in the baseline model was 100 nm, and the magnetic strength was considered as 1.5 T. As larger tumors are more dangerous due to the possibility of disease recurrence after treatment [1,2], in this study a large tumor with a radius of 10 mm was selected.

**Figure 9:**
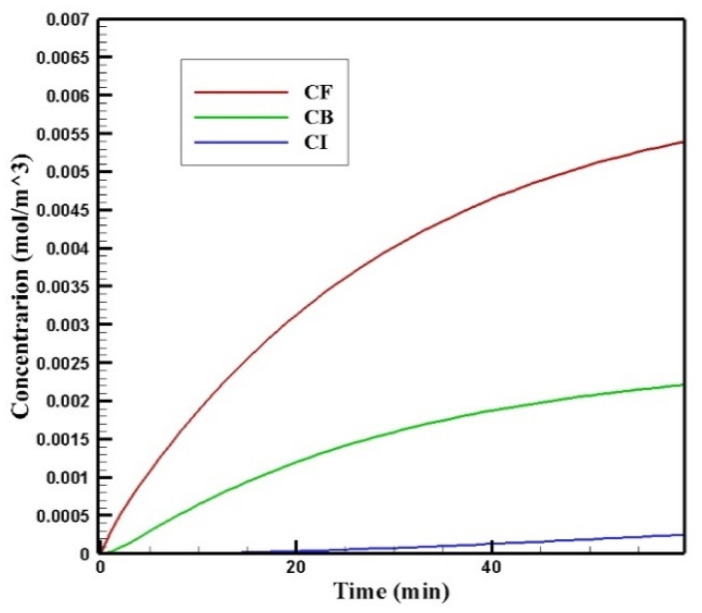
Time profile of the mean concentrations of the free, bound, and internalized drug over 60 min of treatment with a magnetically controlled IP drug targeting system.

**Figure 10:**
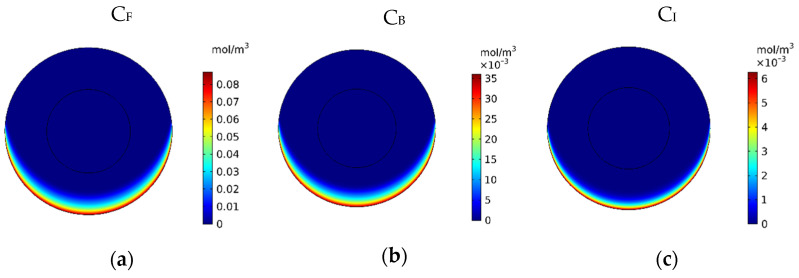
Contours of the (**a**) free, (**b**) bound and (**c**) internalized drug concentrations 60 min after the start of treatment with a magnetically controlled IP drug targeting system.

In Figure 9, the mean concentrations of the free, bound, and internalized drugs are presented over 60 min of treatment. At the end of the treatment, *C_F_*, *C_B_,* and *C_I_* were calculated to be 0.0054, 0.0022, and 0.00025, respectively. Compared to the corresponding values for conventional IPC (i.e., 0.0012, 0.0005, and 0.009), an increase of 4.5, 4.4, and 2.77 times in the mean concentration of the free, bound, and internalized drug were achieved, respectively. Contours of *C_F_*, *C_B_,* and *C_I_* are shown in Figure 10a–c. Comparison of these contours with the contours of conventional IPC in Figure 7 shows that the drug distribution became much better in all of its three forms using MNPs. In traditional intraperitoneal drug delivery, the drug penetrates the outer border of the tumor uniformly from all radial directions. But in drug delivery using a magnet, the magnetic force determines the area where the drug enters the tumor. In the case we simulated here, the direction of the magnetic force was upward. Thus, in the lower area of the tumor, the magnetic force pushed the drug into the tumor tissue, while in the upper area of the tumor, it the opposite effect and impeded the drug from penetrating the tumor.

**Figure 11:**
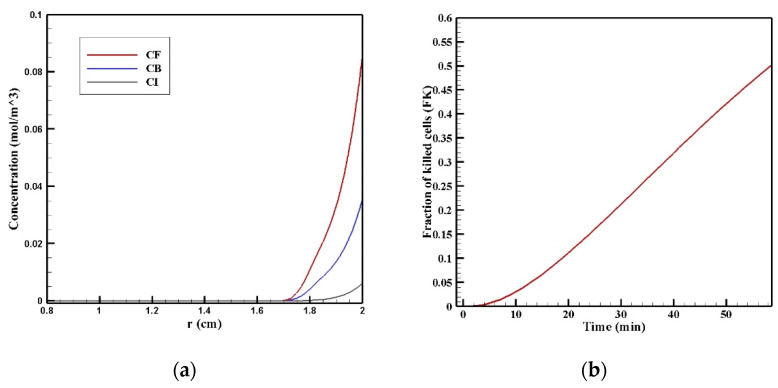
(**a**) Diagram of the mean concentrations of free (*C_F_*), bound (*C_B_*), and internalized drug (*C_I_*) after 60 min; (**b**) killed cells fraction (FK) during 60 min of chemotherapy with intraperitoneal injection.

In Figure 11a, the *C_F_*, *C_B_*, and *C_I_* distributions along the vertical line passing through the center of the tumor are shown 60 min after the start of injection. The drug had a relatively good penetration into the tumor at the lower area of the tumor (*r* = 2 cm), the concentration of the drug decreased in all of its three forms as we moved to the center of the tumor. Figure 11b presents the fraction of killed cells in the area with high drug penetration (*FK_PA_*) over time. *FK_PA_* gradually increased from zero at the starting point to 51.2% at the end of the treatment. For more accurate evaluation and comparing the efficacy of treatment using MNPs, *w*_1/2_, *PA_rel_*, *FK_PA_*, and *FK_eff_* were calculated and presented in Table 5. *w*_1/2_ was equal to 0.08 cm using magnetic nanoparticles with a radius of 100 nm, which shows the significant effect of the proposed technique in increasing the penetration depth of the drug by more than 13 times. As mentioned above, the penetration of the drug in the magnetic nanoparticles’ drug delivery occurred only in the lower area of the tumor. Moreover, the value of the drug penetration area in the magnetic drug delivery was equal to 0.40 cm^2^, which, compared to conventional IPC, showed an increase of more than 1.4 times. The value of *FK_ef_*_f_ in the tumor using the magnetic drug delivery was also calculated to be 6.5%, which shows a more than 2.5 times increase than the obtained value for the conventional method (2.54%). Therefore, considering all these criteria for the treatment’s evaluation, it can be said that magnetically controlled IP drug targeting has improved drug delivery to the tumor compared to the conventional IPC using doxorubicin. The performance of the proposed method was also compared with the results of two other drugs including paclitaxel and cisplatin (Supplementary Materials Figure S1). The average concentration of paclitaxel and cisplatin 60 min after the treatment were 0.0013 and 0.0006 mol/m^3^, respectively, all of which were less than the amount obtained for magnetically controlled drug delivery. Therefore, using this method can also be useful for the transfer of other types of drugs. In the following, by changing the controllable parameters, such as the size of the MNPs, magnetic strength, and the magnet’s distance from the tumor, drug delivery was studied to achieve higher treatment efficacy using this technique.

#### 3.2.2. Effect of the Magnetic Nanoparticles’ Size

**Figure 12:**
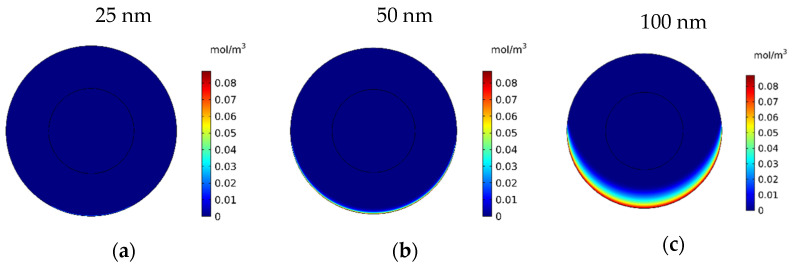

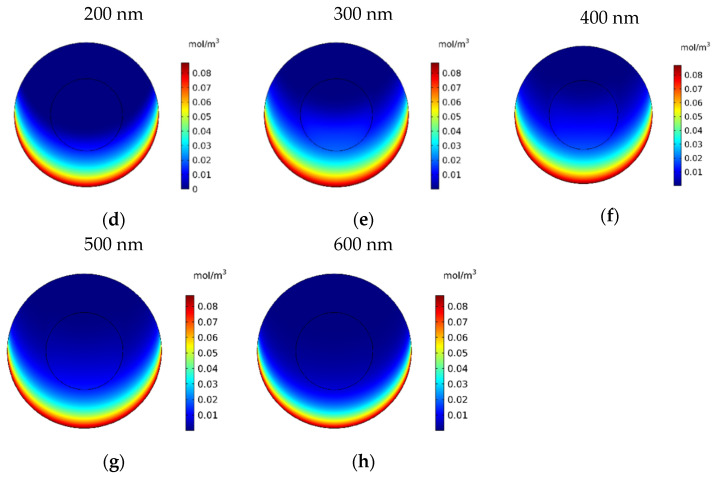
Contours of the free drug concentration (*C_F_*) after 60 min of treatment with magnetically controlled IP chemotherapy for different nanoparticle radius: (**a**) 25 nm, (**b**) 50 nm, (**c**) 100 nm, (**d**) 200 nm, (**e**) 300 nm, (**f**) 400 nm, (**g**) 500 nm, and (**h**) 600 nm.

In this section, the impact of the size of the MNPs used in magnetically controlled IP chemotherapy was studied. Eight different nanoparticles with radii in the range of 25–600 nm were studied. In Figure 12a–h, contours of the free drug concentration distribution are shown for radius ranges of 25, 50, 100, 200, 300, 400, 500, and 600 nm. These contours indicate that magnetic nanoparticles with radii of 25 and 50 nm have poor penetration into the tumor, while increasing the size of nanoparticles to 100 nm significantly increased the penetration of the drug from the lower part of the tumor. Increasing the radius of the nanoparticles to a value greater than 100 nm will dramatically improve the penetration of the drug into the tumor.

**Figure 13:**
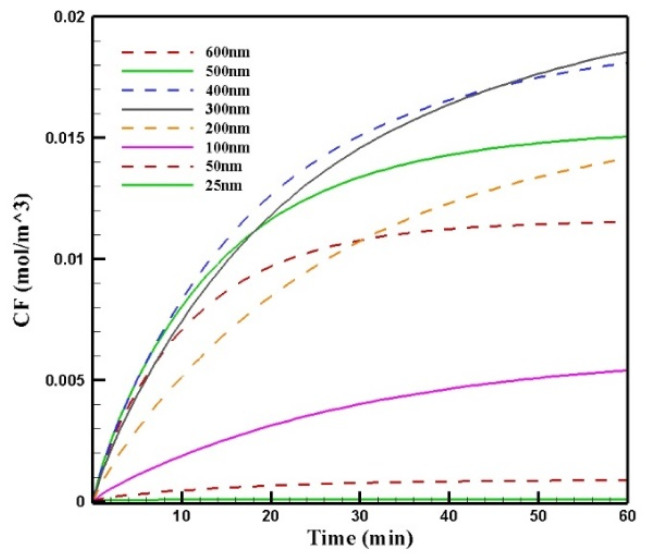
Comparison of the mean concentrations of the free drug (*C_F_*) in the tumor over 60 min of treatment using magnetically controlled IP chemotherapy for different nanoparticle sizes.

Figure 13 shows the mean concentration of free drugs in the tumor for different sizes of MNPs. Comparing the diagrams, we can see that by increasing the size of the MNPs with a radius in the range of 25–300 nm, *C_F_* increased. The change in the size from 300 to 400 nm and to more than 400 nm had different effects on *C_F_*. In this stage of the treatment, the increase in the size of MNPs from 300 to 400 nm resulted in a negligible decrease in the concentration; in contrast, increasing the nanoparticles’ size to more than 400 nm led to a considerable reduction in the concentration of the free drug in the tumor. This was due to the competition of two factors in the penetration of nanoparticles: the magnetic force was greater for larger MNPs, while the microenvironmental barriers of the tumor that impeded the nanoparticles’ transport in the tissue had much more of an effect on the larger particles. Thus, the competition between these two factors, namely, the magnetic force exerted on the particle and the microenvironmental barriers, determined the distribution of MNPs with different sizes.

**Figure 14:**
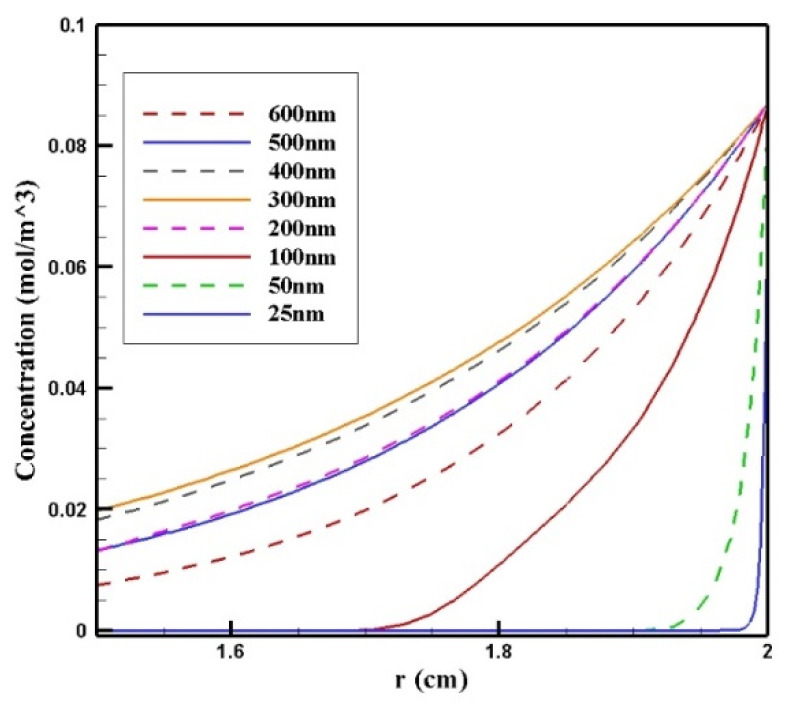
Comparison of the mean concentration of free drug (*C_F_*) along the diameter of the tumor in the area with the maximum penetration depth after 60 min of treatment for different sizes of MNPs.

In Figure 14, *C_F_* distribution along the vertical line of the tumor is presented. Drug delivery using nanoparticles with a radius of 300 and 400 nm resulted in deeper penetration of the drug into the tumor and, subsequently, it increased *C_F_* in the tumor. However, 25 and 50 nm particles showed poor drug penetration into the tumor.

**Figure 15:**
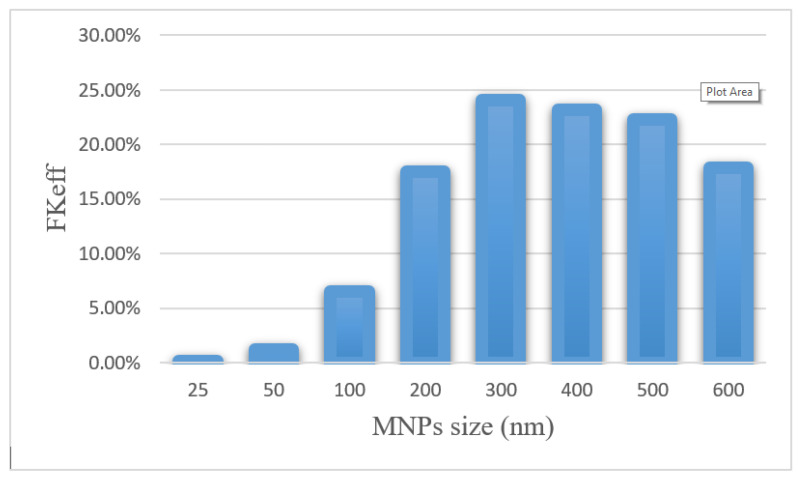
Comparison of *FK_eff_* for different sizes of MNPs after 60 min of treatment.

Efficacy parameters *w*_1/2_, *PA_rel_*, *FK_PA_,* and *FK_eff_* are reported in Table 6. There was an increase in *w*_1/2_ values from 0.003 cm for 25 nm particles to 0.235 cm for 300 nm particles. As the nanoparticle size further increased, *w*_1/2_ decreased, until it reached 0.143 for 600 nm particles. The smallest *PA_rel_* among the compared nanoparticles was related to 25 nm nanoparticles (0.20%), and 300 nm nanoparticles with 55.02% had the largest *PA_rel_* among the studied MNPs. *FK_eff_* values for different sizes of MNPs after 60 min of treatment are presented in Figure 15. *FK_eff_* was calculated as the lowest value for 25 nm nanoparticles, which was equal to 0.11%, whereas it was the highest for 300 nm particles with 24.1%.

The results of this section show that the size of nanoparticles can affect the efficiency of treatment using this method. By selecting an appropriate MNP size, the effective killed cells fraction can increase by 9.5 times compared with conventional IPC. Whereas an improper selection of nanoparticle size (in the range of 25–100 nm) leads to a reduction in the treatment efficacy even lower than the conventional IPC. Additionally, it should be noted that experimental results [51] demonstrate that nanoparticles with a larger size have a longer residence in the peritoneal cavity and will be available to the tumor for a longer time.

#### 3.2.3. The Influence of Magnetic Strength (*B_rem_*)

**Figure 16:**
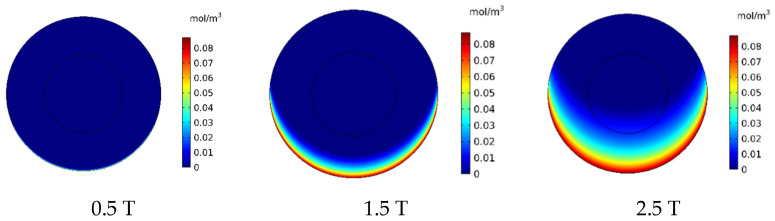
Comparison of the mean concentration of free drug (*C_F_*) in the tumor after 60 min of treatment for different applied magnetic strengths.

In this section, the effect of magnetic strength by changing the remnant magnetic flux (*B_rem_*) in the range of 0.5–2.5 T on the treatment output is investigated. In Figure 16, the contours of *C_F_* distribution in the tumor are presented for the three magnetic strengths of 0.5, 1.5, and 2.5 T. As can be seen, an increase in the magnetic strength will increase the drug’s penetration into the tumor. At the lowest magnetic strength (0.5 T), the drug penetration into the tumor was negligible, whereas at the highest magnetic strength (2.5 T), the drug particles significantly penetrated the tumor.

**Figure 17:**
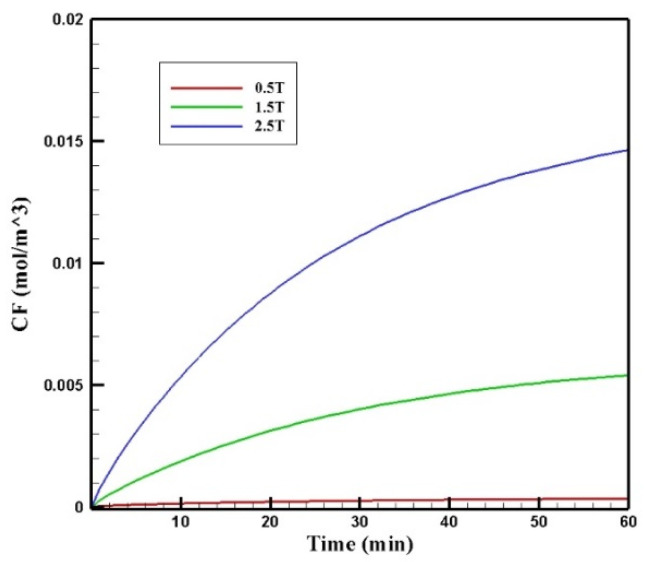
Comparison of the mean *C_F_* values in the tumor over 60 min of treatment for different values of magnetic strengths.

Figure 17 presents the mean *C_F_* profiles over time for different values of magnetic strengths. The mean free drug concentration 60 min after the injection for the three magnetic strengths of 0.5, 1.5, and 2.5 tesla is calculated as 0.00035, 0.00540, and 0.01464 mol/m^3^, respectively. For magnetic strengths of 1.5 and 2.5 tesla, there was an increase in the mean *C_F_* by 4.5 and 12.2 times, respectively. While for the magnetic strength of 0.5 tesla, *C_F_* was 70% less than the corresponding value for conventional IPC.

Table 7 presents the efficacy parameters *w*_1/2_, *PA_rel_*, *FK_PA_,* and *FK_eff_*. According to the results, the penetration depth was 0.006 cm for the magnetic strength of 0.5 tesla, which was less than the corresponding value for the conventional method. For the magnetic strengths of 1.5 and 2.5 tesla, the values obtained for the penetration depth of the drug into the tumor were equal to 0.071 and 0.192 cm, respectively. This indicates an increase of 11.8 and 32 times in penetration depth for the magnetic strengths of 1.5 and 2.5, respectively. In addition, the *PA_rel_* value for the magnetic strength of 0.5 was 0.95%, which is less than the calculated *PA_rel_* for the conventional IPC. Meanwhile, the calculated values for larger magnetic strengths were 12.82% and 38.32% for the magnetic strengths of 1.5 and 2.5 tesla, which showed a 1.43 and 4.2 times increase compared to the conventional IPC, respectively.

**Figure 18:**
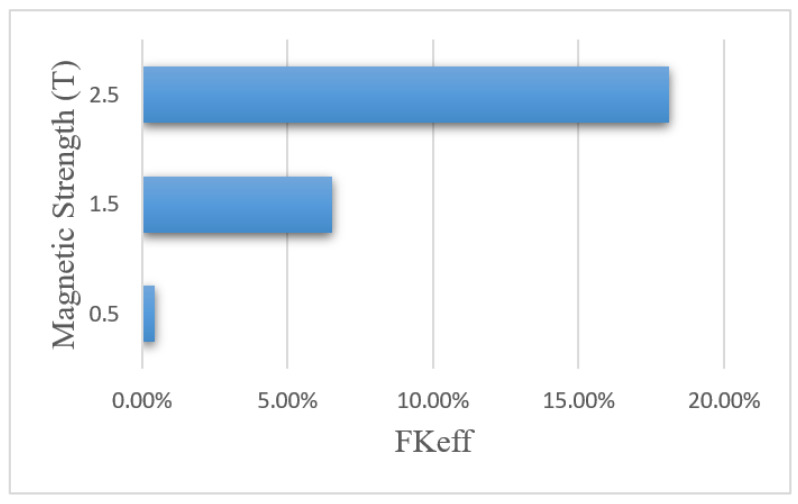
Comparison of *FK_eff_* for different magnet strengths after 60 min of treatment.

Figure 18 shows the *FK_eff_* values for different magnetic strengths after 60 min of treatment. The *FK_eff_* values obtained for the three magnetic strengths of 0.5 tesla, 1.5 tesla, and 2.5 tesla were 0.43%, 6.52%, and 18.1%, respectively. Similar to other efficacy parameters, *FK_eff_* for the magnetic strength of 0.5 tesla was lower than the conventional method, while for the two larger magnetic strengths, it showed an increase by more than 2.5 and 7 times, respectively. This was due to the competition between the magnetic force exerted on the nanoparticles and the microenvironmental barriers, which means that the magnetic force was not large enough to overcome the microenvironmental barriers of the tumor at lower magnetic strengths. The results of this section show that increasing the magnetic strength improves the penetration of the MNPs into the tumor by increasing the magnetic force exerted on the nanoparticles.

#### 3.2.4. The Effect of Tumor Distance from the Magnet

The distance of the permanent magnet from the tumor is another parameter that can influence the performance of the magnetically controlled IP drug targeting system. In reality, the tumor may be located in different sites of the peritoneum area. Thus, the distance between the magnet and the tumor may vary depending on the tumor’s location. Therefore, it is essential to examine the effect of distance and the extent of its impact on the treatment output. In this section, by considering three different distances between the tumor and the permanent magnet (L), this parameter’s effect on the treatment using MNPs was studied.

**Figure 19:**
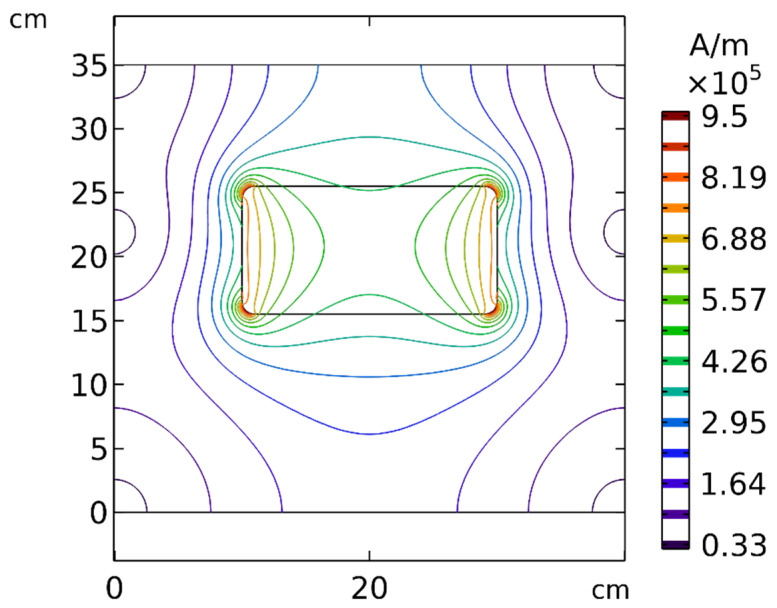
Contours of the magnetic field (*H*) around the permanent magnet and in the solution area.

According to Equation (7), the magnetic force exerted on the MNPs was commensurate with $\nabla^{2}H$, revealing the importance of the strength and gradient of the magnetic field. To better understand the effect of distance, the spatial distribution of the magnetic field around the permanent magnet is presented in Figure 19. As evident in this figure, by increasing the distance between the tumor and the magnet, the strength and the gradient of the magnetic field decreased significantly, which indicates that the distance of the magnet from the tumor can affect the efficiency of the magnetically controlled IP.

**Figure 20:**
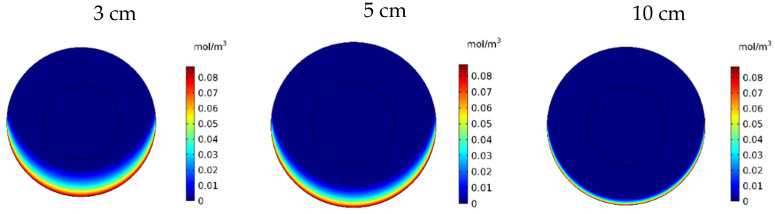
Free drug concentration (*C_F_*) contours 60 min after IP injection using drug-coated MNPs at different distances between the magnetic source and the tumor center.

**Figure 21:**
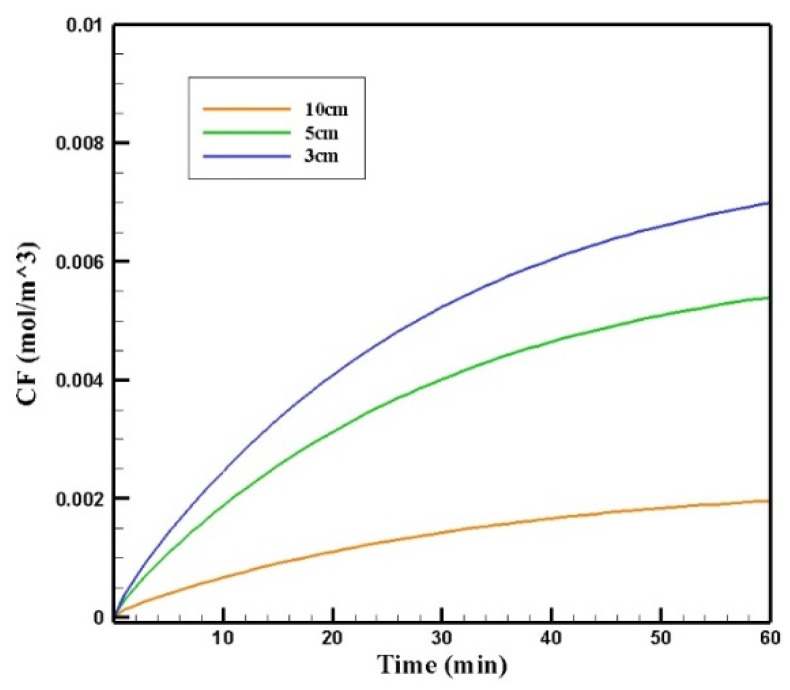
The comparison of the mean concentrations of the free drug (*C_F_*) in tumors over 60 min treatment of IP chemotherapy using drug-coated magnetic nanoparticles at different distances between the magnetic source and the tumor center.

Figure 20 shows the contours of *C_F_* in the tumor for three different distances of the permanent magnet from the tumor including 3, 5, and 10 cm. The comparison of the contours shows that by increasing the distance, a smaller part of the tumor was affected by the drug. This reduction in penetration occurred dramatically, especially by increasing the distance of the magnet from the tumor to 10 cm. According to Figure 21, *C_F_* values 60 min after the injection at a distance of 3 cm was 0.0069 mol/m^3^, while increasing the distance to 10 cm decreased the *C_F_* value to 0.0019 mol/m^3^.

**Figure 22:**
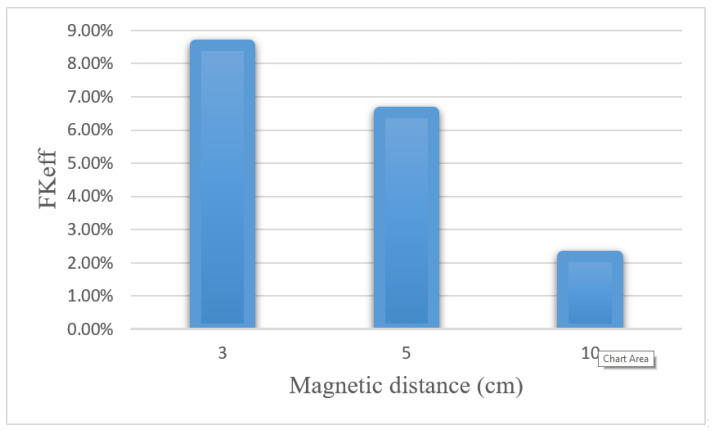
Comparison of *FK_eff_* for different magnetic distances after 60 min of treatment.

Table 8 presents the values obtained for *w*_1/2_, *PA_rel_*, *FK_PA_,* and *FK_eff_* for three distances between the magnet and the tumor after 60 min of treatment. Reducing the distance of the magnet from the tumor from 10 to 3 cm increased the *w*_1/2_ and *PA_rel_* values by 4 and 4.4 times, respectively. Figure 22 compares the *FK_eff_* values for different magnetic distances after 60 min of treatment. *FK_eff_* increased by 3.88 times by reducing the distance of the magnet from the tumor from 10 to 3 cm.

According to the results of this section, changing the distance between the tumor and the magnet significantly affected the efficiency of the magnetically controlled IP chemotherapy. Considering that tumors may be located in different sites of the peritoneal cavity, treatment using MNPs will be more difficult for tumors in deeper parts of the body. Although, using optimal values for other parameters, such as magnetic nanoparticle size and magnetic strength, can help to improve drug delivery to these tumors.

### 3.3. Validation

In this section, the validation of the numerical solution is presented. The IFP and IFV distributions were calculated using the Darcy law and, subsequently, the concentration distribution of the free, bound, and internalized drugs could be obtained by solving the mass transfer equations. Hence, each of these physics must be validated separately.

#### 3.3.1. Validation of the IFP and IFV Distribution

**Figure 23:**
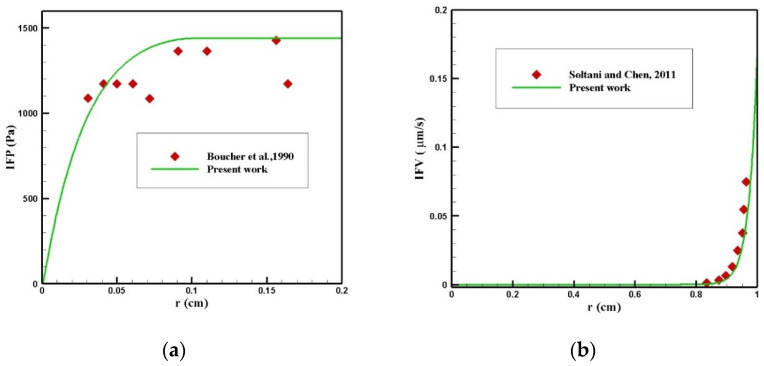
Validation of interstitial fluid flow modeling: (**a**) IFP and (**b**) IFV.

Finding the IFP and IFV is one of the exigent steps of this modeling, which was obtained by solving Equation (1). To validate the distribution of IFP in the tumor, a comparison between the radial distribution of IFP with an experimental work by Boucher et al. [52] was conducted under the same conditions, which is presented in Figure 23a. As can be seen in this figure, there was a good agreement between the values obtained from the modeling and the experimental results. In addition, the values of IFV distribution along the tumor radius were compared with the theoretical values presented by Soltani and Chen [42] (Figure 23b), and there was good agreement between the values of these two graphs.

#### 3.3.2. Validation of the Concentration Distribution

**Figure 24:**
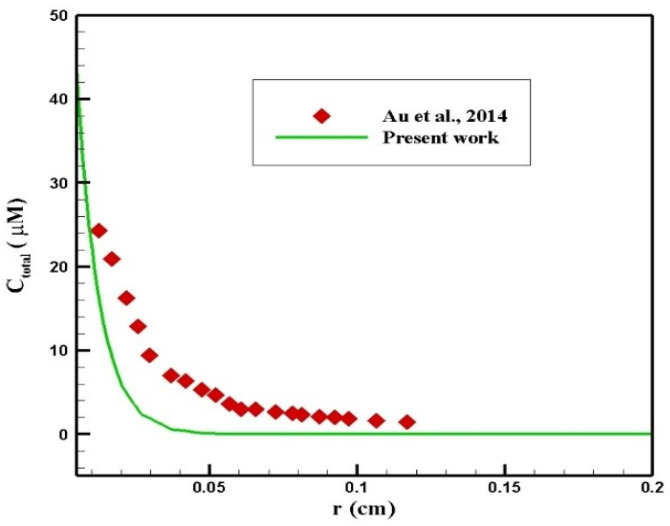
Comparison of the concentration values in terms of penetration depth at 6 h after injection with the values obtained from the study by Au et al. [50].

Au et al. [50] evaluated the performance of chemotherapy by IP injection on a mouse. In their study, the radius of the tumor was 2 mm, and 45 mM of paclitaxel was injected intraperitoneally. A comparison of the concentration profiles in terms of penetration depth within 6 h after injection is presented in Figure 24. As can be seen, there was a relatively good agreement between the two profiles. The difference between the concentration values in the two graphs resulted from different properties of the tissue and the drug in the two studies.

## 4. Conclusions

In the present study, a mathematical model was developed to study a magnetically controlled IP drug targeting system as a solution to improve the drug penetration into the tumor. Considering the binding and internalization of the drug into the cancer cells, the mathematical model analyzed the drug distribution inside the tumor in three forms: free, bound, and internalized. The drug penetration area and the fraction of killed cells in the tumor were used to estimate the efficiency of the proposed drug delivery system. The main conclusions of the present study are as follows:

1. Using a magnetically controlled IP drug targeting system can overcome the microenvironmental barriers of the tumor against the transfer of drug particles and significantly increase the treatment efficiency compared to conventional IP chemotherapy;
2. There was an optimal size for MNPs: larger nanoparticles exerted a stronger magnetic force. On the other hand, tumor microenvironmental barriers further hinder the movement of large nanoparticles in the tissue compared to smaller ones. Therefore, the competition between these two factors (i.e., the magnetic force to the particle and the hindering effects of the microenvironmental barriers) determines the extent of penetration of different sizes of nanoparticles;
3. Using a permanent magnet with more magnetic strength and reducing the magnet’s distance from the tumor by increasing the magnetic force exerted on the MNPs improved IP delivery of doxorubicin-coated MNPs.

There are a number of assumptions and limitations in our model. With the lack of appropriate experimental data associated with the drug concentration at the outer edge of the tumor, this value was assumed constant during the simulation. By sampling the intraperitoneal fluid during treatment, a more accurate boundary condition for concentration outside the tumor can be obtained. In addition, the tissue poroelasticity effect was not considered in this study. This effect should be considered in the future developments of the model, given that studies have shown the effect of these phenomena on drug binding and internalization [53]. The parameters used in our model were derived from prior experimental investigations that were consistent with the numerical studies too. We adopted the spherical symmetric approximation assuming that the infusion occurs within a circular domain. Although the present study does not claim to be a replacement for experimental studies, the mathematical model of magnetically controlled IP drug targeting is a powerful tool that can help us gain insights into the impacts of various parameters.

## Figures and Tables

**Table 1:** Parameters for tumor tissue.

Parameter	Definition	Unit	Value	References
S/V	Surface area of blood vessels per unit tissue volume	m^−1^	2 × 10^4^	[42,43]
k	Hydraulic conductivity of the interstitium	m^2^·Pa^−1^·s^−1^	3 × 10^−14^	[44,45]
$L_{P}$	Hydraulic conductivity of the microvascular wall	m·Pa^−1^·s^−1^	2.10 × 10^−11^	[44]
$P_{B}$	Vascular fluid pressure	Pa	2.1 × 10^3^	[42]
$\pi_{B}$	Osmotic pressure of the plasma	Pa	2.7 × 10^3^	[28]
$\pi_{i}$	Osmotic pressure of the interstitial fluid	Pa	2 × 10^3^	[28]
$\sigma_{s}$	Average osmotic reflection coefficient for plasma proteins	-	0.9	[44]

**Table 2:** Solute transport parameters used in the simulation.

Parameter	Definition	Unit	Value	References
$D_{eff}$	Effective diffusion coefficient	cm^2^·s^−1^	3.40 × 10^−6^	[46]
P	Microvessel permeability coefficient	cm·s^−1^	3.00 × 10^−4^	[46]
$K_{ON}$	Constant of the binding rate	M^−1^·s^−1^	1.5 × 10^2^	[25,47,48]
$K_{OFF}$	Constant of the unbinding rate	s^−1^	8 × 10^−3^	[25,47,48]
$K_{INT}$	Constant of the cell uptake rate	s^−1^	5 × 10^−5^	[25,47,48]
φ	Tumor volume fraction accessible to drugs	-	0.3	[9]
$C_{rec}$	Concentration of cell surface receptors	M	1 × 10^−5^	[25]
ω	Cancer cell survival constant	m^3^·mol^−1^	0.4938	[40]

**Table 3:** Boundary conditions employed for the present model.

Boundary Conditions		Region
Concentration	Fluid Flow	
$(D_{F}\nabla C+\upsilon_{i}C)\lfloor \Omega^{-}=\left(D_{F}\nabla C+\upsilon_{i}C\right)\lfloor \Omega^{+}$ $C\lfloor \Omega^{-}=C\lfloor \Omega^{+}$	$-k\nabla P_{i}\lfloor \Omega^{-}=-k\nabla P_{i}\lfloor \Omega^{+}$ $P_{i}\lfloor \Omega^{-}=P_{i}\lfloor \Omega^{+}$	The inner boundary of the tumor
C=Constant	$P_{i}=Constant$	The outer boundary of the tumor

**Table 4:** Treatment evaluation parameters after 60 min of treatment with intraperitoneal injection by the traditional method.

Evaluation Parameter	$\left(w_{1/2}\right)$	*PA_rel_*	*FK_PA_*	*FK_eff_*
Value	0.006 cm	8.97%	28.3%	2.54%

**Table 5:** Calculated values of treatment efficacy parameters after 60 min of treatment using an IP injection in drug delivery, which employed 100 nm magnetic nanoparticles.

$w_{1/2}$	*PA_rel_*	*FK_PA_*	*FK_eff_*
0.08 cm	12.73%	51%	6.5%

**Table 6:** Comparison of treatment efficacy parameters for different sizes of MNPs after 60 min of treatment.

Nanoparticle Size (nm)	$w_{1/2}$	*PA_rel_*	*FK_PA_*	*FK_eff_*
25	0.003 cm	0.20%	56.3%	0.11%
50	0.011 cm	2.90%	43.4%	1.25%
100	0.071 cm	12.82%	50.8%	6.52%
200	0.18 cm	36.51%	47.9%	17.5%
300	0.235 cm	55.02%	43.8%	24.1%
400	0.221 cm	54.81%	42.3%	23.2%
500	0.18 cm	55.00%	40.5%	22.3%
600	0.143 cm	44.24%	40.5%	17.9%

**Table 7:** Comparison of the calculated values of the treatment efficacy parameters for different magnetic strengths applied for a nanoparticle size of 100 nm after 60 min of IP injection.

Magnetic Strength (T)	$\left(w_{1/2}\right)$	*PA_rel_*	*FK_PA_*	*FK_eff_*
0.5	0.006 cm	0.955%	45.8%	0.43%
1.5	0.071 cm	12.827%	50.8%	6.52%
2.5	0.192 cm	38.324%	47.3%	18.1%

**Table 8:** Comparison of calculated values of treatment efficacy parameters at different distances between the magnetic source and the center of the tumor 60 min after IP injection.

Magnet Distance	$\left(w_{1/2}\right)$	*PA_rel_*	*FK_PA_*	*FK_eff_*
3 cm	0.096 cm	17.44%	48.9%	8.54%
5 cm	0.071 cm	12.82%	50.8%	6.52%
10 cm	0.024 cm	3.94%	56.5%	2.2%

## Data Availability

The data presented in this study are available on request from the corresponding author.

## Supplementary Materials

The following supporting information can be downloaded at: https://www.mdpi.com/article/10.3390/pharmaceutics14020324/s1, Figure S1: The average drug concentration in the tumor for conventional IP chemotherapy using Doxorubicin, Paclitaxel, and Cisplatin, compared with the result of magnetically controlled IP drug delivery during 60 minutes of treatment.
