# Supplementary material for: Mathematical Modeling of Targeted Drug Delivery Using Magnetic Nanoparticles during Intraperitoneal Chemotherapy
Source: Pharmaceutics. 2022 Jan 29;14(2):324. doi: 10.3390/pharmaceutics14020324 (PMC8875578; doi:10.3390/pharmaceutics14020324)
Supplement: Supplementary file 1 [file pharmaceutics-14-00324-s001.zip › pharmaceutics-1506408-supplementary.pdf]

Supplementary Materials: Mathematical Modeling of Targeted Drug Delivery Using Magnetic Nanoparticles during Intraperitoneal Chemotherapy

Mohsen Rezaeian [1], M. Soltani [1,2,3,4,*], Ahmad Naseri Karimvand [1] and Kaamran Raahemifar [5,6,7]

1 Department of Mechanical Engineering, K. N. Toosi University of Technology, Tehran 470, Iran; mohsenrezaeian@email.kntu.ac.ir (M.R.); ahmad.Naserik999@gmail.com (A.N.K.)
2 Centre for Biotechnology and Bioengineering (CBB), University of Waterloo, Waterloo, ON N2L 3G1, Canada
3 Department of Electrical and Computer Engineering, University of Waterloo, Waterloo, ON N2L 3G1, Canada
4 Advanced Bioengineering Initiative Center, Multidisciplinary International Complex, K. N. Toosi University of Technology, Tehran 470, Iran
5 Data Science and Artificial Intelligence Program, College of Information Sciences and Technology (IST), Penn State University, State College, Pennsylvania, PA 16801, USA; kvr5517@psu.edu
6 Department of Chemical Engineering, University of Waterloo, 200 University Avenue West, Waterloo, ON N2L 3G1, Canada
7 School of Optometry and Vision Science, Faculty of Science, University of Waterloo, 200 University Avenue West, Waterloo, ON N2L 3G1, Canada
* Correspondence: msoltani@uwaterloo.ca; Tel./Fax: +1-(519)-888-4567

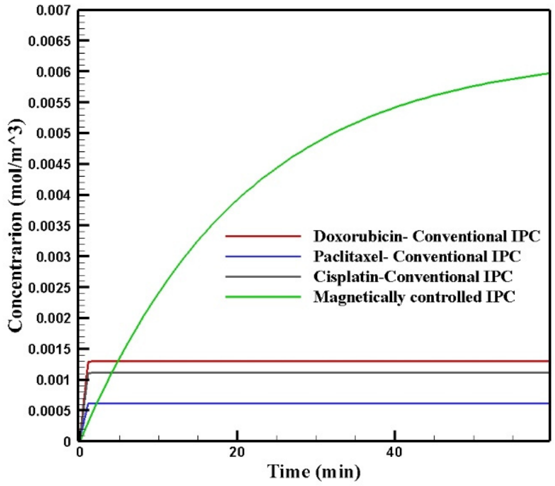

Figure S1: The average drug concentration in the tumor for conventional IP chemotherapy using Doxorubicin, Paclitaxel, and Cisplatin, compared with the result of magnetically controlled IP drug delivery during 60 min of treatment.
